# Throat and rectal swabs to diagnose melioidosis: utility or futility?

**DOI:** 10.1128/jcm.00113-26

**Published:** 2026-05-14

**Authors:** Robert C. Duguid, Robert W. Baird, Jann Hennessy, Bart J. Currie, Ella M. Meumann

**Affiliations:** 1 Department of Paediatrics, Royal Darwin Hospital 71507, Darwin, Australia; 2 Microbiology Department, Territory Pathology, Royal Darwin Hospital 71507, Darwin, Australia; 3 Department of Infectious Diseases, Division of Medicine, Royal Darwin Hospital 71507, Darwin, Australia; 4 Global and Tropical Health Division, Menzies School of Health Research and Charles Darwin University 10095, Darwin, Australia; Mayo Clinic, Rochester, Minnesota, USA

**Keywords:** melioidosis

## LETTER

Melioidosis, caused by *Burkholderia pseudomallei,* remains a major cause of sepsis in tropical regions, with diagnosis largely reliant on culture from clinical samples (1). The sensitivity of throat swabs for melioidosis diagnosis has been reported to be 24–36%, while that of rectal swabs is 16%; culture of *B. pseudomallei* from any site is considered significant, and specificity is reported to be 100% (2–4). In the ‘Top End’ of the Northern Territory (NT), Australia, guidelines recommend throat and rectal swab culture for patients with suspected melioidosis, alongside culture of blood and other clinically relevant specimens (5). Due to the wide range of melioidosis presentations, it is challenging to predict clinically which patients have melioidosis in our setting, meaning that testing is extensive (6).

We evaluated the performance of throat and rectal swabs for *B. pseudomallei* culture over a 10-year period (1 October 2014 to 30 September 2024) at Royal Darwin Hospital, the tertiary center serving the NT Top End. We linked laboratory culture data with the Darwin Prospective Melioidosis Study database, which includes all culture-confirmed melioidosis cases in the region (6). After swabbing the throat or rectum, cotton swabs were placed in Ashdown’s broth and incubated for 5 days, with subculture to solid media if turbidity or a pellicle was observed (Supplemental materials). *B. pseudomallei* was identified as previously described (7, 8).

During the study period, the laboratory processed 17,826 throat swabs and 18,413 rectal swabs for *B. pseudomallei* culture, with median 1,821 (interquartile range [IQR]: 1,635–1,838) throat and 1,847 (IQR: 1,701–1,919) rectal swabs per annum. Overall positivity was low: 0.8% (143/17,826) for throat swabs and 0.3% (51/18,413) for rectal swabs. After de-duplication to include only one result per patient per site in a 7-day period, positivity for throat swabs was 0.7% (114/16,937) and 0.3% (45/17,561) for rectal swabs. Among 595 confirmed melioidosis cases, 114 of 450 (25.3%) with a throat swab collected had *B. pseudomallei* isolated, and 45 of 455 (9.9%) with a rectal swab. Negative predictive values were 97.8% (16,601/16,937) and 97.4% (17,151/17,561), respectively. Among patients with melioidosis pneumonia, 99 of 275 (36.0%) had a positive throat swab compared to 15 of 175 (8.6%) in those with melioidosis without pneumonia (*P* ≤ 0.0001). A total of 36 of 277 (13%) patients with pneumonia had a positive rectal swab compared to 14 of 203 (6.9%) without pneumonia (*P* = 0.03). The sensitivity of throat and rectal swab culture in patients with pneumonia and those without pneumonia with other primary presentations is presented in Table 1. Detection in throat swabs from those without pneumonia likely represents subclinical involvement of the respiratory tract. Detection inrectal swabs may result from swallowed sputum or gastrointestinal tract involvement.

**TABLE 1:** Proportion of melioidosis patients with pneumonia (primary or secondary) with *B. pseudomallei* isolated from throat and/or rectal swabs and those without pneumonia with other primary presentations

Clinical syndrome	No. of patients	Throat swab positive/no. of swabs (%)	Throat swab exclusive diagnosis no. (%)	Rectal swab positive/no. of swabs (%)	Rectal swab exclusive diagnosis no. (%)
Pneumonia	367	99/275 (36.0%)	5 (1.8%)	36/277 (13.0%)	3 (1.1%)
Skin abscess	70	3/55 (5.5%)	1 (1.8%)	0/54 (0.0%)	0 (0.0%)
Genitourinary infection	54	5/44 (11.4%)	0 (0.0%)	5/45 (11.1%)	0 (0.0%)
Bacteremia without focus	32	2/27 (7.4%)	0 (0.0%)	2/28 (7.1%)	0 (0.0%)
Soft tissue infection	25	0/17 (0.0%)	0 (0.0%)	0/19 (0.0%)	0 (0.0%)
Septic arthritis	10	0/4 (0.0%)	0 (0.0%)	0/4 (0.0%)	0 (0.0%)
Osteomyelitis	9	2/6 (33.3%)	0 (0.0%)	0/6 (0.0%)	0 (0.0%)
Central nervous system infection	2	0/2 (0.0%)	0 (0.0%)	0/2 (0.0%)	0 (0.0%)
Other site	26	3/20 (15.0%)	2 (10%)	7/45 (15.6%)	0 (0.0%)
Total	595	114/450 (25.3%)	8 (1.8%)	45/455 (9.9%)	3 (0.7%)

Importantly, positive throat or rectal swabs were rarely the sole diagnostic specimen. There were eight (8/450, 1.8%) cases where only a throat swab was culture positive, three (3/455, 0.7%) where only a rectal swab was positive, and one where both throat and rectal swabs but no other specimens were positive (Table S1). These 12 cases were treated for melioidosis solely on the basis of these swab results. Among these cases, clinical review raised uncertainty about causality in several rectal swab-only diagnoses, where the site of positivity was distant from the apparent focus of disease, and biological plausibility was not clear (Supplemental materials).

Our findings highlight a mismatch between laboratory workload and diagnostic yield; culture of throat and rectal swabs for all patients with suspected melioidosis offers limited benefit. Throat swabs remain of value in suspected pulmonary melioidosis, particularly when sputum cannot be obtained, including in children. We plan to limit rectal swab culture to second-line testing, when initial investigations are non-diagnostic, in patients with high clinical probability of melioidosis. This diagnostic stewardship approach is unlikely to compromise patient outcomes.

## AFTER EPUB

*[This article was published on 14 May 2026 with an error in funding information. The funding information was updated in the current version, posted on 28 May 2026.]*

## SUPPLEMENTAL MATERIAL

Supplemental materials jcm.00113-26-s0001.pdf Detailed methods.

ASM does not own the copyrights to Supplemental Material that may be linked to, or accessed through, an article. The authors have granted ASM a non-exclusive, world-wide license to publish the Supplemental Material files. Please contact the corresponding author directly for reuse.
