# Supplementary material for: Throat and rectal swabs to diagnose melioidosis: utility or futility?
Source: J Clin Microbiol. 2026 May 14;64(6):e00113-26. doi: 10.1128/jcm.00113-26 (PMC13251406; doi:10.1128/jcm.00113-26)
Supplement: Supplemental materials — Detailed methods. [file jcm.00113-26-s0001.pdf]

Supplementary Appendix

Methods

Study design and population

The study was conducted in the Microbiology Department at Royal Darwin Hospital, the tertiary referral centre for the “Top End” of the Northern Territory (NT), using data from a 10-year period from 1 October 2014 to 30 September 2024. The Darwin Prospective Melioidosis Study (DPMS) has documented the clinical and epidemiological characteristics of all culture-confirmed cases of melioidosis in the Top End of the Northern Territory, Australia since 1989.[1] Definitions used in the DPMS for clinical presentations were as defined previously. The DPMS has the same catchment area as the laboratory. Several private pathology providers serve primary care centres in the region; occasionally *B. pseudomallei* is isolated in these laboratories, but subsequent patient management is through Royal Darwin Hospital. The DPMS therefore represents a complete dataset for notified melioidosis cases in the Top End region.

The study was approved by the Human Research Ethics Committee of the Northern Territory Department of Health and Menzies School of Health Research (approval number 02/38).

Microbiological methods

Ashdown’s agar and broth are selective media useful for the diagnosis of melioidosis from non-sterile sites.[2-3] After swabbing the throat or rectum, cotton swabs are inoculated into 10 mL Ashdown’s broth (TM1156, Thermo Fisher Scientific) by the collecting health practitioner; the wooden swab stick is broken off and remains in the broth. On arrival in the laboratory, the broth cultures are incubated aerobically at 35°C for 5 days. Broths are examined daily, and if there is turbidity and/or pellicle formation (Figure S1) they are subcultured onto Ashdown’s agar (PP2200, Thermo Fisher Scientific). Ashdown’s plates are incubated at aerobically at 35°C and are examined at 24 and 48 hours. Broth cultures are not subcultured if they remain clear at day 5. Until recently, identification of *B. pseudomallei* was conducted using automated biochemical testing with the Vitek 2 GN ID card and confirmation by qPCR targeting the *B. pseudomallei* type III secretion system.[4] Since 2024, MALDI-TOF MS using the Vitek MS with v3.3 of the knowledge base (bioMérieux) has been used in place of automated biochemical testing.[5]

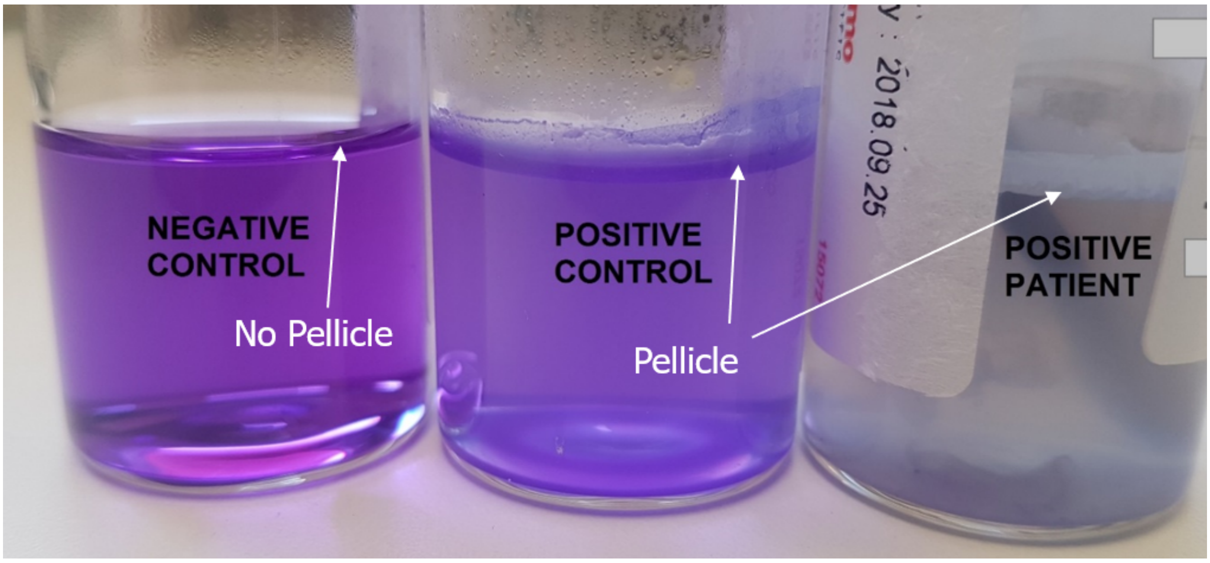

Figure S1. Ashdown's broth with pellicle formation.

Data analysis

Data cleaning, linkage, and analysis were performed in R (version 2025.05.1+513; R Foundation for Statistical Computing, Vienna, Austria).

Throat and rectal swabs collected 7 days before or after the case diagnosis date were linked to melioidosis episodes in the DPMS. A maximum of one positive or one negative swab from each site (throat and/or rectal) were included, with other swabs up to 90 days after the diagnosis date excluded; 90 days was selected as this is the minimum duration of therapy for melioidosis. The proportion of patients with each clinical syndrome with positive throat and rectal swabs was calculated. Patients were classified as having pneumonia (either as a primary or secondary diagnosis) or by their primary clinical syndrome if they did not have pneumonia as either a primary or a secondary diagnosis.

The sensitivity of throat and rectal swabs was calculated as the proportion of culture-confirmed melioidosis patients (with a swab collected) with a positive swab. To calculate negative predictive value, we identified all throat and rectal swabs collected within the study period that were not linked to any DPMS episode within 7 days before or after the collection date; these were considered true negative swabs. These true negative swabs were de-duplicated to one per patient and site within a 7-day period. False negatives were negative swabs from patients with confirmed melioidosis within 7 days either side of their melioidosis diagnosis date. 95%

confidence intervals for sensitivity and negative predictive values were calculated using the Wilson method.

Cases with no positive *B. pseudomallei* culture from any other site but with a positive throat and/or rectal swab were classified as swab-exclusive diagnoses. The clinical histories of these cases were reviewed. If *B. pseudomallei* was noted to have been isolated from another clinical specimen at an external laboratory, these were not included as swab-exclusive diagnoses.

Description of unique cases

Of the eight patients with throat swabs as their unique diagnostic sample, five (40%) had pneumonia (Table S1). One paediatric patient had erythema nodosum. Other cases included adults with chronic mandibular osteomyelitis, non-healing lower limb ulcers and pharyngitis with erythema nodosum. None of the eight patients had a sputum sample collected.

There were three cases of melioidosis where a rectal swab was the unique diagnostic sample (Table S1). Case 1 was an adult patient with diabetes and chronic kidney disease, who had a typical presentation with acute community-acquired pneumonia resulting in septic shock and intensive care unit admission. They had multiple negative blood cultures, and no sputum sample was collected. The other two cases were unusual. Case 2 was an adult patient with diabetes whose positive rectal swab result was only noted after they were discharged following treatment for community acquired pneumonia with ceftriaxone (an agent with limited activity against *B. pseudomallei*). They were subsequently treated for melioidosis 6 months later during an admission with pancreatitis, but did not have clinical evidence of melioidosis at that time and *B. pseudomallei* was not isolated. Case 3 was an adult with diabetes and chronic kidney disease who was diagnosed with melioidosis one month into an admission and treatment for *Enterococcus faecalis* bacteremia. The patient had a fever without clinical focus and returned a positive rectal swab, so was treated for melioidosis. They were found to have consolidation on chest X-ray and a skin abscess, but did not have sputum collected for culture and did not culture *B. pseudomallei* from the skin lesion.

There was one patient who had both throat and rectal swabs that were positive for *B. pseudomallei* without additional sites of culture positivity. This patient was a 13-year-old who presented with erythema nodosum and was subsequently found to have pulmonary nodules on CT chest, though was asymptomatic of these and no sputum sample was sent.

Table S1. Unique throat and rectal swab melioidosis diagnoses

Age/sex	Clinical syndrome	Rectal swab	Throat swab	Sputum
27/M	Chronic mandibular osteomyelitis, pneumonia	Negative	Positive	Not collected
47/F	Pneumonia	Negative	Positive	Not collected
7/F	Erythema nodosum	Negative	Positive	Not collected
71/M	Erythema nodosum	Negative	Positive	Not collected
34/M	Pneumonia	Negative	Positive	Not collected
32/F	Skin abscess	Negative	Positive	Not collected
88/F	Pneumonia	Negative	Positive	Not collected
79/F	Pneumonia, osteomyelitis	Negative	Positive	Not collected
47/M	Pneumonia	Positive	Negative	Not collected
54/M	Pneumonia	Positive	Negative	Not collected
60/F	Pneumonia, skin abscess	Positive	Negative	Not collected
13/F	Erythema nodosum, pulmonary nodules	Positive	Positive	Not collected

M=male; F=female

References

1. Currie BJ, Mayo M, Ward LM, et al. The Darwin Prospective Melioidosis Study: a 30-year prospective, observational investigation. *Lancet Infect Dis*. 2021;21(12):1737-1746. doi:10.1016/S1473-3099(21)00022-0
2. Dance DAB, Sihalath S, Rith K, et al. The cost-effectiveness of the use of selective media for the diagnosis of melioidosis in different settings. *PLoS Negl Trop Dis*. 2019;13(7):e0007598. Published 2019 Jul 15. doi:10.1371/journal.pntd.0007598
3. Wuthiekanun V, Dance DA, Wattanagoon Y, Supputtamongkol Y, Chaowagul W, White NJ. The use of selective media for the isolation of Pseudomonas pseudomallei in clinical practice. *J Med Microbiol*. 1990;33(2):121-126. doi:10.1099/00222615-33-2-121
4. Novak RT, Glass MB, Gee JE, et al. Development and evaluation of a real-time PCR assay targeting the type III secretion system of *Burkholderia pseudomallei*. *J Clin Microbiol*. 2006;44(1):85-90. doi:10.1128/JCM.44.1.85-90.2006
5. Campbell S, Taylor B, Menouhos D, et al. Performance of MALDI-TOF MS, real-time PCR, antigen detection, and automated biochemical testing for the identification of *Burkholderia pseudomallei*. *J Clin Microbiol*. 2024;62(10):e0096124. doi:10.1128/jcm.00961-24
